# Facets of Theiler’s Murine Encephalomyelitis Virus-Induced Diseases: An Update

**DOI:** 10.3390/ijms20020448

**Published:** 2019-01-21

**Authors:** Ingo Gerhauser, Florian Hansmann, Malgorzata Ciurkiewicz, Wolfgang Löscher, Andreas Beineke

**Affiliations:** 1 Department of Pathology, University of Veterinary Medicine, Bünteweg 17, 30559 Hannover, Germany; Ingo.Gerhauser@tiho-hannover.de (I.G.); Florian.Hansmann@tiho-hannover.de (F.H.); Malgorzata.Ciurkiewicz@tiho-hannover.de (M.C.); 2 Center for System Neuroscience, 30559 Hannover, Germany; Wolfgang.Loescher@tiho-hannover.de; 3 Department of Pharmacology, University of Veterinary Medicine, Bünteweg 17, 30559 Hannover, Germany

**Keywords:** adaptive immune response, animal model, demyelination, epilepsy, innate immune response, multiple sclerosis, myocarditis, seizures, Theiler’s murine encephalomyelitis virus

## Abstract

Theiler’s murine encephalomyelitis virus (TMEV), a naturally occurring, enteric pathogen of mice is a Cardiovirus of the Picornaviridae family. Low neurovirulent TMEV strains such as BeAn cause a severe demyelinating disease in susceptible *SJL* mice following intracerebral infection. Furthermore, TMEV infections of *C57BL/6* mice cause acute polioencephalitis initiating a process of epileptogenesis that results in spontaneous recurrent epileptic seizures in approximately 50% of affected mice. Moreover, *C3H* mice develop cardiac lesions after an intraperitoneal high-dose application of TMEV. Consequently, TMEV-induced diseases are widely used as animal models for multiple sclerosis, epilepsy, and myocarditis. The present review summarizes morphological lesions and pathogenic mechanisms triggered by TMEV with a special focus on the development of hippocampal degeneration and seizures in *C57BL/6* mice as well as demyelination in the spinal cord in *SJL* mice. Furthermore, a detailed description of innate and adaptive immune responses is given. TMEV studies provide novel insights into the complexity of organ- and mouse strain-specific immunopathology and help to identify factors critical for virus persistence.

## 1. General Aspects of Theiler’s Murine Encephalomyelitis Virus

**Figure 1:**
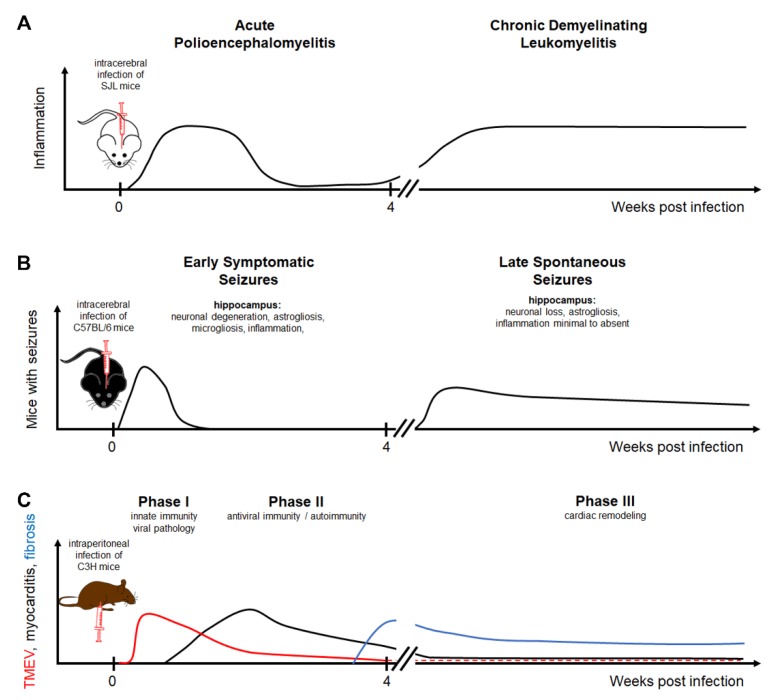
Theiler’s murine encephalomyelitis virus infection as a model for multiple sclerosis, epilepsy, and myocarditis: (**A**) The intracerebral infection of *SJL* mice with TO subgroup strains of TMEV results in a biphasic disease course consisting of an acute polioencephalomyelitis followed by a chronic demyelinating leukomyelitis. (**B**) The intracerebral infection of *C57BL/6* mice leads to early symptomatic seizures in the acute phase associated with neuronal degeneration, astrogliosis, microgliosis, and inflammation in the hippocampus. Following a latent phase, mice can develop spontaneous seizures associated with neuronal loss and astrogliosis in the hippocampus. (**C**) The intraperitoneal infection of *C3H* mice leads to an infection of the heart. The acute phase is divided into phase I characterized by viral pathology triggering innate immunity and phase II consisting of antiviral immune responses and autoimmunity inducing myocardial damage. Cardiac remodeling is a dominant feature of phase III. TMEV strains are divided into two major groups, a highly neurovirulent group called George Davis 7 (GDVII) consisting of GDVII and FA strains and a low neurovirulent group called Theiler’s original (TO) including the *BeAn 8386* (BeAn), *Daniels* (DA), *TO4*, *WW*, *Yale*, and *4727* strains [1,24,25,26]. Highly neurovirulent TMEV strains induce an acute, severe, frequently fatal polioencephalitis, while low neurovirulent strains induce a biphasic disease with acute polioencephalomyelitis and subsequent chronic, progressive, demyelinating leukomyelitis [1,3,27,28].

Theiler’s murine encephalomyelitis virus (TMEV)-induced diseases are used as models for multiple sclerosis (MS), virus-induced seizures/epilepsy, and virus-induced myocarditis [1,2,3,4,5,6,7,8,9]. The virus belongs to the genus Cardiovirus of the Picornaviridae family, which also includes Vilyuisk human encephalomyelitis virus (VHEV), Theiler’s-like virus of rats, Saffold virus, and Saffold virus 2 [10]. TMEV is a rodent pathogen and induces mild gastroenteritis following oronasal infection while the infection of the central nervous system (CNS) is a rare event [3,11,12,13]. Experimental intracerebral infection of young adult mice causes flaccid paralysis of the hind limbs [3,7]. The course of Theiler’s murine encephalitis (TME) depends on the virus and mouse strains as well as other factors including intestinal microbiota and stress [3,14,15,16,17]. Mice develop an acute polioencephalomyelitis within two weeks following experimental intracerebral TMEV infection. However, the genetic background of mice in addition to the virus strain has an important impact on the disease course, especially on the development of chronic demyelination (Figure 1). Referring to TMEV-induced demyelinating disease (TMEV-IDD), mouse strains can be divided into three different categories: (i) highly susceptible strains including *SJL*, *DBA/1*, *DBA/2*, *SWR*, *PL*, and *NZW* strains; (ii) intermediately susceptible strains including *C3H*, *CBA*, *AKR*, and *C57BR* strains, and (iii) resistant strains including *BALB/c*, *C57BL/6*, *C57BL/10*, *C57/L*, and *129/J* [18]. While resistant mouse strains eliminate the virus from the CNS within a few weeks after infection, mouse strains developing TMEV-IDD show virus persistence in the spinal cord associated with progressive demyelinating leukomyelitis [12,14,19,20,21,22]. Nevertheless, *C57BL/6* mice resistant to chronic demyelination are prone to develop early (encephalitis-associated) seizures and chronic epilepsy, whereas mouse strains susceptible for demyelination, especially *SJL* do not develop seizures and epilepsy [19,23].

The genome of TMEV has a size of approximately 8100 nucleotides, which codes for a polyprotein with approximately 2300 amino acids. Translation of the polyprotein is regulated by an internal ribosomal entry site (IRES). This polyprotein is cleaved into 12 proteins including L, Viral proteins 1–4 (VP1–4), 2A–C, and 3A–D [29,30]. VP1–4 act as basic structures of the viral capsid while 2A and 3C represent proteases, 2C represents a nucleoside-triphosphatase, 3B represents a binding site for IRES, and 3D represents a *RNA*-dependent RNA polymerase [28,31]. The functions of viral proteins 2B and 3A are still unknown. However, investigations using poliovirus indicate that these proteins interfere with the function of interleukin (IL) 6 and IL8 and interferon (IFN) β secretion. Furthermore, proteins 3A and 2B may inhibit tumor necrosis factor (TNF) α-mediated apoptosis or promote apoptosis, respectively [32,33,34]. Virus transcription is regulated by many factors including cellular microRNAs as well as long noncoding RNAs (lncRNAs). While cellular microRNAs seem to have no significant impact on TMEV replication, an enhancer-like lncRNA, termed NeST which interacts with the murine viral susceptibility locus *Tmevp3* and thereby controls IFNγ expression, is involved in TMEV persistence and may serve as a potential target for novel antiviral drugs [35,36].

## 2. Virus Tropism and Spread

TMEV tropism depends on the viral capsid structure which facilitates neuronal infections by highly neurovirulent strains and infections of glial cells and macrophages by low neurovirulent strains [37,38,39,40,41,42]. Highly neurovirulent TMEV strains are suggested to bind to heparan sulfate, while low neurovirulent strains are shown to target sialic acids [43]. Nonetheless, the cellular entry receptor for TMEV is still undetermined [43,44]. Highly neurovirulent strains infect higher cell numbers than low neurovirulent strains but produce similar levels of viral RNA, indicating that their more efficient spread is probably not related to a higher replication rate [45]. In the acute disease phase, all TMEV strains primarily replicate in neurons, whereas in the chronic phase, virus replication takes place primarily in astrocytes in TMEV-BeAn-infected *SJL* mice and in oligodendrocytes in TMEV-DA-infected *C.B-17* mice [46,47,48]. Cell culture studies using recombinant viruses of high and low neurovirulent strains demonstrated that the amount of virus production in astrocytes, macrophage/microglial cells, oligodendrocytes, and oligodendrocyte precursor cells is strongly dependent on the P1 capsid region [41].

TMEV has a specific tropism for the CA1 and CA2 pyramidal cell layers of the hippocampus; periventricular thalamic nuclei; septal nuclei; and piriform, parietal, and entorhinal cortices during acute TMEV infection [49,50,51]. The majority of virus antigens in and around chronic demyelinating lesions can be found in macrophages, which take up viral particles by phagocytosis. However, virus replication in these cells is limited [47,52,53]. Similarly, in vitro studies demonstrate a restricted virus replication in TMEV-infected microglia, whereas astrocytes produce high amounts of viral RNA and antigen [54]. The induction of apoptosis represents a mechanism to restrict virus replication because TMEV virions are cleaved by executioner caspases, such as caspase-3, disassembling virions into pentamers [55]. Astrocytes are relatively resistant to TMEV-induced apoptosis, whereas neurons are predominantly affected by apoptotic cell death during acute polioencephalomyelitis [41,56,57]. The causes of apoptosis resistance of glial cells are not known in detail, but survivin and the overexpression of caspase-1 (formerly IL1β converting enzyme (ICE)) seems to be involved in the resistance of astrocytes to programmed cell death [58,59]. The infection of resident CNS cells entails the activation of innate and subsequent adaptive immune responses, which are needed for the restriction of virus replication but also result in severe bystander damage and immunopathology during acute and chronic infection.

Virus spread from the brain to the spinal cord can take place via the axonal and/or hematogenous routes [60]. The demonstration of ependymal infections and subjacent spread to the parenchyma around the fourth ventricle also indicates a liquorogenic dissemination [60]. An important hallmark of TME is axonal degeneration and loss [61]. In general, axonal degeneration follows different mechanisms: (i) primary axonal damage due to disruption/dysregulation of axonal transport (inside out) or (ii) axonal degeneration following demyelination [61,62]. In TME, the inside-out mechanism due to TMEV-induced neurofilament accumulation as well as the outside-in mechanism characterized by demyelination leading to glial cell loss followed by increased axonal vulnerability and loss have been observed [61,62]. Although causing clinical disability, axonal degeneration might represent a beneficial self-destructive process to reduce TMEV spread via fast axonal transport [46,63,64]. 

## 3. Theiler’s Murine Encephalomyelitis Virus Infection as Model for Multiple Sclerosis

**Figure 2:**
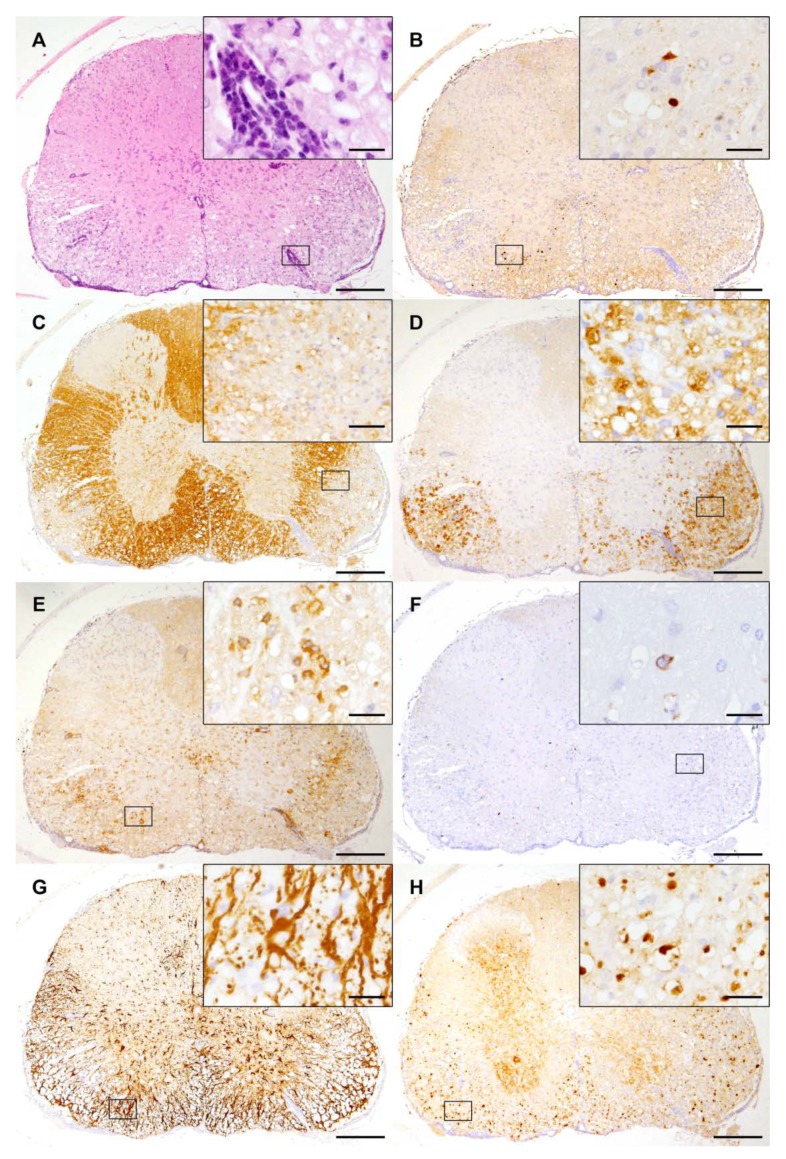
Spinal cord lesions induced by Theiler’s murine encephalomyelitis virus in *SJL* mice: (**A**) Spinal cord lesions at 14 weeks post infection consist of mononuclear meningomyelitis centered in the ventral white matter (hematoxylin eosin staining). (**B**) The immunohistochemistry of spinal cord lesions reveals virus protein mainly located in the ventrolateral funiculus. (**C**) Demyelination is illustrated by a loss of myelin basic protein staining. (**D**) Myelin debris is removed by activated microglia/macrophages (MAC3/CD107b). (**E**) Abundant T cells (CD3) infiltrate spinal cord lesions. (**F**) Only low numbers of B-lymphocytes (CD45R) are present in the TMEV-infected spinal cord. (**G**) Astrogliosis (GFAP) with numerous activated astrocytes is present in spinal cord lesions. (**H**) Axonal degeneration is characterized by the accumulation of non-phosphorylated neurofilaments and spheroid formation. (**A**–**H**) The inserts show higher magnifications of the areas delineated by the black rectangles. The bars represent 200 µm and 20 µm for overviews and inserts, respectively.

TMEV-IDD of susceptible mouse strains, such as the *SJL* mice, is a well-established animal model for the progressive forms of MS [65,66,67,68]. The etiology of MS is still unknown, but an infectious, inflammatory, degenerative, and/or immune-mediated disease is suspected [69]. Hallmarks of MS and TME are inflammation, demyelination, axonal degeneration, and astrogliosis (Figure 2) [65]. Recent TME studies investigating biomarkers in the CNS and cerebrospinal fluid of mice identified CCL5 (RANTES), CXCL9 (MIG), and CXCL10 (IP10) as being strongly upregulated in the chronic phase, which resembles chemokine expression patterns observed in MS patients [70]. Intracerebral infection of susceptible mice with low neurovirulent TMEV strains leads to a biphasic disease with acute polioencephalomyelitis followed by chronic progressive, demyelinating leukomyelitis starting at about 28 days post infection (Figure 2) [3]. 

Despite a high similarity of amino acid sequences between low neurovirulent TMEV strains, DA strains induce more pronounced lesions compared to BeAn strains [71,72,73,74]. During the acute phase of TME, predominantly neurons within the hippocampus and the cerebral cortex are infected. In the chronic phase, TMEV lesions are characterized by lymphohistiocytic meningomyelitis with perivascular infiltrates, demyelination, astrogliosis, and axonal degeneration as well as axonal loss, predominantly in the ventral part of the spinal cord [75,76,77,78]. Representative spinal cord lesions induced by TMEV are shown in Figure 2. TMEV is predominantly located in glial cells including oligodendrocytes, astrocytes, and microglia [20,31]. TMEV-infected oligodendrocytes may undergo virus-induced apoptosis or destruction by microglia/macrophages and cytotoxic T cells leading to a release of viral antigens and myelin epitopes [47,79]. Apoptotic oligodendrocytes can already be detected in the pre-demyelinating phase (14 days post infection) in the spinal cord white matter of *SJL* mice infected with TMEV-BeAn, demonstrating that oligodendroglial apoptosis might contribute to the initiation of myelin loss [57]. 

**Figure 3:**
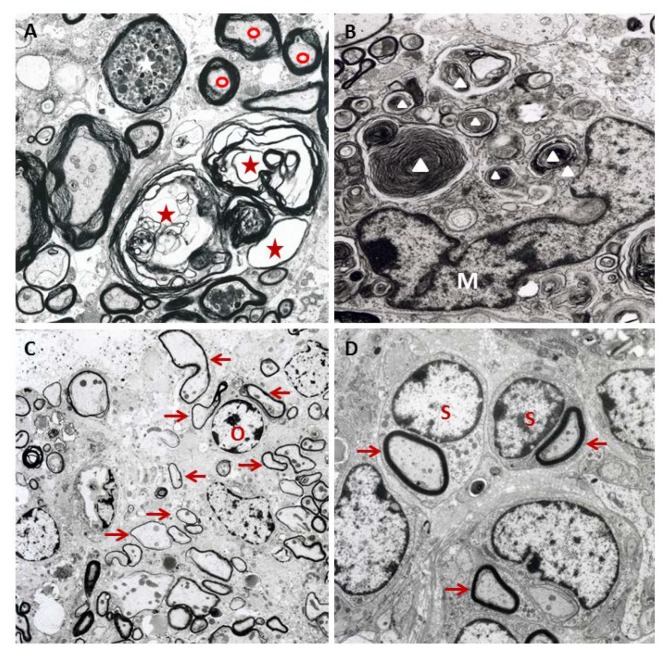
The ultrastructure of spinal cord lesions in Theiler’s murine encephalomyelitis virus-infected *SJL* mice: (**A**) A dilated myelin sheath (red asterisk) with an irregular separation of myelin lamellae (intramyelinic vacuolization) and axon loss, a degenerated axon with an accumulation of electron dense material (white asterisk) at 42 days post infection (dpi), and a normally myelinated axon (red circle) are shown. (**B**) Macrophages/microglia with phagocytized myelin fragments (white triangles) at 42 dpi, characteristic of myelinophagia (M = nucleus of a macrophage/microglial cell), are shown. (**C**) A demyelinated lesion containing remyelinated axons with thin myelin sheaths (arrows), characteristic of oligodendrocyte-mediated remyelination (O = nucleus of an oligodendrocyte), is shown at 196 dpi. (**D**) Schwann cell remyelination at 196 dpi, characterized by comparatively thick newly formed myelin sheaths (arrows) and a one Schwann cell per axon relationship (S = nucleus of a Schwann cell), is shown.

Initial T cell responses are directed against viral antigens. During the chronic phase, the development of type IV hypersensitivity leads to demyelination and axonal damage [80,81]. The liberation of myelin compounds in damaged foci induces immune responses targeting proteolipid protein (PLP), as well as myelin basic protein (MBP) and myelin oligodendrocyte glycoprotein (MOG) due to epitope spreading [82,83]. Molecular mimicry between viral and myelin epitopes has been discussed as a mechanism leading to the development of an autoimmune process against oligodendrocytes [84,85]. Matrix metalloproteinase 12 plays a crucial role for the progression of demyelination since this enzyme exhibits oligodendrocyte- and myelin-toxic properties and is involved in macrophage extravasation [77]. Despite the presence of intra- and perilesional oligodendrocyte precursor cells and the onset of remyelination by oligodendrocytes and Schwann cells in the chronic phase [86], neuroregeneration is incomplete in TMEV-IDD [78,87]. Factors leading to disturbed remyelination include the downregulation of cholesterol biosynthesis, astrogliosis, and extracellular matrix accumulation (e.g., collagen IV, laminin, perlecan, tenascin-C, collagen I, decorin, entactin, neurocan, and fibronectin) [76,86,87]. Characteristic ultrastructural changes of demyelination and remyelination processes are illustrated in Figure 3.

## 4. Theiler’s Murine Encephalomyelitis Virus Infection as Model for Seizures and Epilepsy

Epilepsy is the most common serious neurological disorder worldwide affecting 70 million people especially in low- or medium-income countries [88]. Moreover, 30% to 40% of patients with epilepsy are non-responders to medications [89]. The disorder is characterized by recurrent unprovoked seizures, which occur after a long process of structural and functional changes in the brain [90]. In addition to genetic causes, epilepsy often results from a head trauma, stroke, brain tumors, and encephalitis [90]. Several viruses including influenza virus, West Nile virus, herpes viruses, and non-polio picornaviruses can cause encephalitis and acute (encephalitis-associated) seizures. Survivors have a high risk of developing chronic epilepsy with spontaneous recurrent seizures and comorbidities, including memory loss [91,92,93,94]. Appropriate animal models of epilepsy induced by viral encephalitis are lacking due to the high mortality of mice following neurotropic virus infections [94]. 

**Figure 4:**
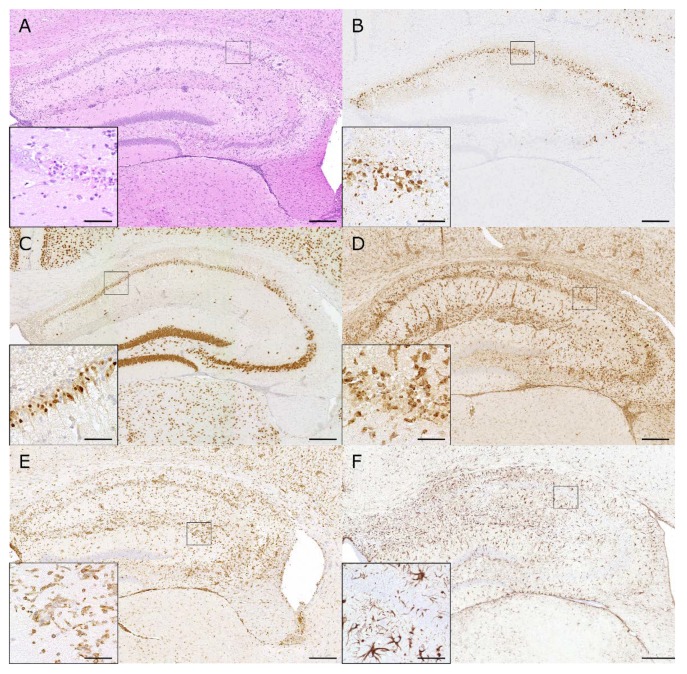
Hippocampal lesions induced by Theiler’s murine encephalomyelitis virus in *C57BL/6* mice: (**A**) Intracerebral infection of *C57BL/6* mice with the DA strain of TMEV causes hippocampal inflammation (HE) and neuronal pyknosis. (**B**) The TMEV antigen is mainly localized in pyramidal neurons of the CA1 and CA2 region at 7 days post infection (dpi). (**C**) Marked pyknosis and loss of NeuN-positive neurons are seen in the hippocampus at 7 dpi. (**D**) Prominent infiltration of macrophages and activation of microglia (MAC3/CD107b) are seen in the hippocampus at 7 dpi. (**E**) Numerous T cells (CD3) are seen in the hippocampal lesions at 7 dpi. (**F**) Neuronal degeneration and inflammatory changes are associated with strong astrogliosis (GFAP) at 14 dpi. (**A**–**F**) The inserts show higher magnifications of the areas delineated by the black squares. The bars represent 200 µm and 20 µm for overviews and inserts, respectively.

Approximately 50% of TMEV-DA-infected *C57BL/6* mice develop transient early (acute) afebrile seizures associated with impaired motor function and coordination within 3 to 7 days after infection followed by a seizure-free period and significantly reduced seizure thresholds months after infection [2,50]. In contrast, TMEV-BeAn-infected *C57BL/6* mice rarely develop seizures and *SJL* mice are completely seizure-resistant, demonstrating virus- and mouse strain-specific mechanisms of ictogenesis during TMEV infection [23]. Early seizures are associated with a virus-mediated decrease in hippocampal CA3 inhibitory network activity [95]. The percentage of *C57BL/6* mice developing early seizures after a TMEV-DA infection is strongly dependent on the inoculated viral dose but does not correlate with viral clearance, which occurs uniformly by day 14 post infection [96]. Pyramidal neuron pyknosis also correlates with early seizure activity in TMEV-DA-infected *C57BL/6* mice and is associated with oxidative stress in the hippocampal area [2,97]. Moreover, *C57BL/6* mice with early seizures show increased perivascular cuffing, astrogliosis, and high numbers of macrophages/microglia in the hippocampus, which is similar to human temporal lobe epilepsy [98,99,100]. Typical hippocampal lesions induced by TMEV are shown in Figure 4.

**Figure 5:**
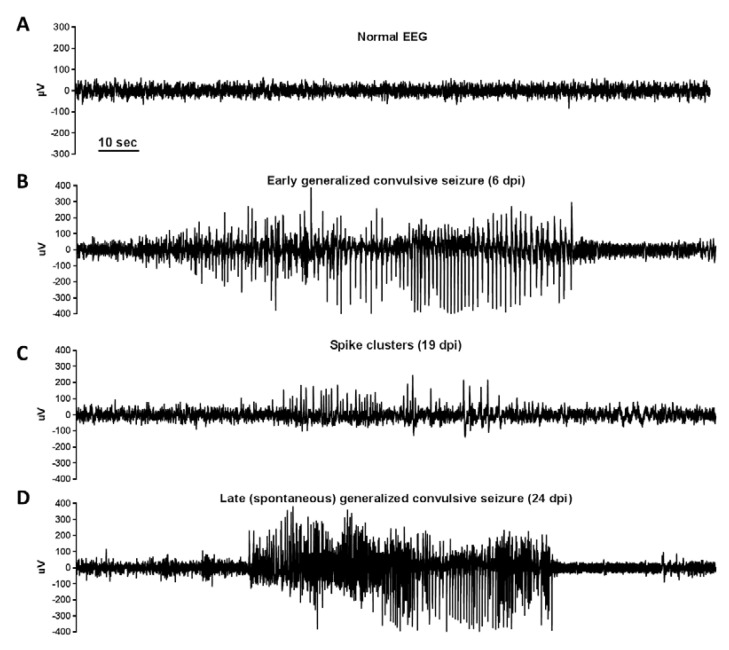
EEG recordings in TMEV-DA-infected *C57BL/6* mice: TMEV-DA-infected *C57BL/6* mice were continuously (24/7) video/EEG monitored for the occurrence of early (0–7 dpi) and late (>7 dpi) seizures. EEG recordings were performed via chronically implanted cortical electrodes. Early seizures were exhibited in 77% of the mice in this study, while the incidence of late spontaneous seizures was 33%. The figure illustrates (**A**) a normal cortical EEG recording, (**B**) an early generalized convulsive seizure, (**C**) spikes and spike clusters in a mouse with epilepsy, and (**D**) a late (spontaneous) generalized convulsive seizure. Based on EEG and video recordings, early and late seizures exhibited the same phenomenological characteristics. However, late epileptic seizures were much less frequent than early seizures (~1 seizure/week vs. 5.5 seizures/week; *p* < 0.05). Furthermore, late generalized convulsive seizures were significantly longer than such seizures in the early phase (1–7 dpi) of monitoring. In addition to generalized convulsive seizures, nonconvulsive (focal) seizures were observed both during the early and late phase following infection. The average latent phase before the onset of late spontaneous seizures was 61 days (range 16–91 days). Reproduced with permission from [101], published by Elsevier, 2018.

*C57BL/6* mice, that display acute (early) symptomatic seizures, also have significantly reduced seizure thresholds and develop a chronic state of hyperexcitability leading to epilepsy with spontaneous recurrent epileptic seizures in approximately 50% of affected mice [4,50]. Moreover, video-electroencephalogram (EEG) monitoring two, four, and seven months after TMEV-DA infection revealed epileptiform EEG activity (i.e., spikes) in 100% of mice, which is associated with severe cell loss in CA1 and CA2 pyramidal cell layers of the hippocampus, astrogliosis, and corresponding enlargement of the lateral ventricles [4]. Likewise, EEG spikes and spike clusters are significantly more frequent in TMEV-DA-infected *C57BL/6* mice with late seizures compared to infected mice without seizures [101]. Typical EEG alterations in TMEV-DA-infected *C57BL/6* mice are shown in Figure 5. Epileptic mice also develop an impaired cognitive ability and anxiety-like behavior [102]. Interestingly, the minocycline treatment of TMEV-infected *C57BL/6* mice demonstrates that acute seizure control alone is insufficient to modify chronic disease comorbidities because administering this anti-inflammatory and neuroprotective drug did not affect the percentage of mice with acute seizures but in fact improved long-term behavioral outcomes and normalized seizure threshold [103]. Consequently, inflammatory processes seem to be involved in the process of epileptogenesis. 

## 5. Theiler’s Murine Encephalomyelitis Virus Infection as Model for Myocarditis

The TMEV infection of TMEV-IDD susceptible mice represents a suitable model for studying virus-mediated myocarditis [5,8,104,105,106,107]. Since this model was recently described in detail elsewhere, it will be introduced only briefly in this review [5,105]. The most effective way of inducing cardiac disease is by the intraperitoneal high-dose injection of TMEV [108]. *C3H* mice are susceptible to the development of cardiac lesions, which are classified into three different phases: phase I consists of viral pathology including type I IFN (IFN-I) and chemokine responses, phase II consists of an uncontrolled antiviral immune response leading to myocardial damage by a bystander process and/or by epitope spreading (autoimmune myocarditis), and phase III is characterized by cardiac remodeling and fibrosis resulting in dilated cardiomyopathy [5,105]. In contrast to *C3H* mice, *C57BL/6* mice show only mild cellular infiltrates in the heart and do not exhibit cardiac fibrosis. *SJL* mice generally show no cellular infiltrates in the heart [5,105]. 

## 6. Innate Immunity in Theiler’s Murine Encephalomyelitis Virus Infection

### 6.1. Mechanisms of the Innate Immune Response

**Figure 6:**
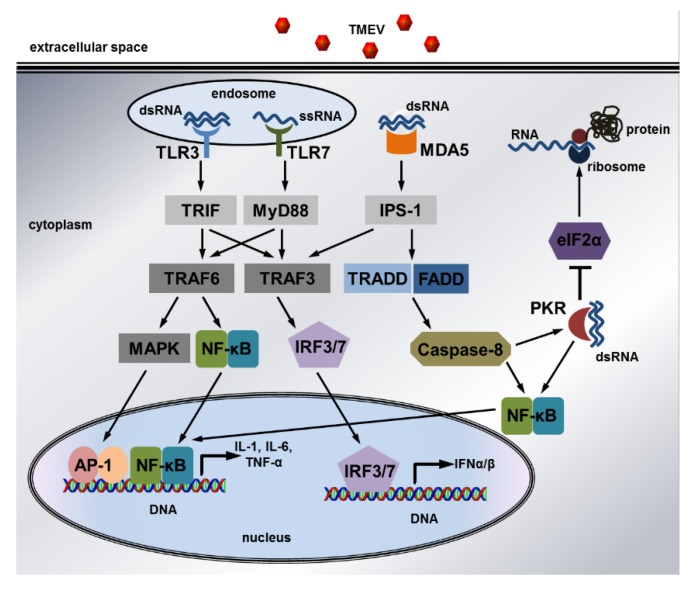
The intracellular signaling pathways induced by Theiler’s murine encephalomyelitis virus: Detection of dsRNA by TLR3 and ssRNA by TLR7 in endosomes leads to the recruitment of TRIF and MyD88, which interact with TRAF3 and TRAF6 to trigger MAPK, NF-κB, and IRF signaling. This results in transcription of the pro-inflammatory cytokines IL1, IL6, and TNFα as well as antiviral IFNα/β. Viral dsRNA in the cytoplasm is recognized by MDA5, which activates IPS-1 to initiate IRF signaling via TRAF3. Moreover, MDA5 triggers NF-κB signaling via TRADD and FADD as well as caspase-8. NF-κB signaling can also be induced by PKR, which is activated by binding to cytoplasmic dsRNA. In addition, PKR phosphorylates eIF2α to inhibit mRNA and viral RNA translation and thus virus replication. Finally, caspase-8 can cleave the inhibitory N-terminus of PKR to activate this antiviral protein, whose expression is stimulated by IFNα/β. Only selected pathway members are shown due to simplification. Solid arrows indicate activation while “T” indicates inhibition.

Cytokines, chemokines, effector molecules, and cells of the innate immune system contribute to the development of adaptive immune responses and TMEV-induced diseases (recently reviewed in References [94,109]). Innate immune responses are activated by specific germline-encoded receptors including Toll-like receptors (TLRs), retinoic acid-inducible gene (RIG)-I-like receptors (RLRs), NOD-like receptors (NLRs), and C-type lectin receptors (CLRs). These pattern recognition receptors (PRRs) recognize structures conserved among microbial species such as lipopolysaccharides (LPS) and viral RNA, which are called pathogen-associated molecular patterns (PAMPs), but also endogenous molecules released from damaged cells, termed damage-associated molecular patterns (DAMPs) [110]. The detection of PAMPs and DAMPs by PRRs leads to the activation of nuclear factor κ-light-chain-enhancer of activated B cells (NF-κB), mitogen-activated protein kinases (MAPKs), and IFN regulatory factors (IRFs), which induce the expression of cytokines, chemokines, and IFN-I including IFNα and IFNβ [110]. Intracellular double-stranded *RNA* (dsRNA) produced during virus infections can also be bound by dsRNA-activated protein kinase (PKR), which prevents virus replication by inhibiting the translation of mRNAs and viral RNAs through the phosphorylation of eukaryotic translation initiation factor 2A (eIF2α) [111]. TMEV-induced intracellular signaling pathways leading to the expression of pro-inflammatory cytokines and IFN-I are summarized in Figure 6.

Cytoplasmic PKR also contributes to immunity against TMEV by regulating the mRNA stability of IFN-I [112]. These signaling proteins are critical mediators of the inflammatory and antiviral immune response directed against TMEV [109]. IFN-I can activate T cells, stimulate B cells, induce NK cell cytotoxicity, and enhance the expression of major histocompatibility complex (MHC) class I molecules and co-stimulatory molecules (CD40, CD80, and CD86) on antigen-presenting cells [113,114,115,116,117,118]. Nevertheless, IFN-I also induces IL10 and reduces the blood-brain-barrier permeability and the IFNγ-induced expression of MHC class II antigen [113,119,120]. These studies demonstrate that the temporal and spatial production of IFN-I has to be strictly controlled in order to take advantage of its antiviral functions while avoiding its negative immunomodulatory effects. The inhibition of IFN-I signaling pathways is essential for the efficient replication of viruses including TMEV [121]. For instance, apolipoprotein L9 is an interferon stimulated gene (ISG), which probably cooperates with cellular prohibitins to restrict the replication of TMEV-DA [122]. TMEV inhibits the production of IFN-I by blocking the nuclear translocation of IRF3 with its L protein [123,124]. The inhibition of nucleocytoplasmic trafficking is caused by L protein-exportin interactions and subsequent phosphorylation of Phe/Gly-containing nuclear pore proteins (Nup62 and Nup98) [125,126]. Furthermore, persistent TO strains express an additional protein called L* protein via an alternative open reading frame (ORF) in the regions of L, VP4, and VP2, which inhibit the IFN-I inducible oligoadenylate synthetases (OAS)/RNase L pathway that cleaves viral and cellular RNA [127,128].

Microglia expression of IFN-I is stimulated not only directly by virus infection but also indirectly by the pro-inflammatory cytokine IFNγ via the activation of IRF-1 [129]. This transcription factor is also involved in T helper (Th) 1 differentiation by inducing IL12 expression in macrophages and by mediating the response of T cells to IL12 stimulation [130,131]. Consequently, TMEV-infected IFNγ^−/−^ *C57BL/6* mice are characterized by the reduced expression of antiviral IFN-I and by impaired protective CD4^+^ and CD8^+^ T cell responses [129,132]. A stronger expression of IFNγ mRNA can also be found in the CNS of TMEV-BeAn-infected *C57BL/6* compared to *SJL* mice, which is probably mediated by NF-κB and contributes to viral elimination [133]. The inhibition of protective, early IFNγ-producing CD4^+^ and CD8^+^ T cell responses in TMEV-BeAn-infected *SJL* mice seems to be caused by an excessive activation of the NLRP3 inflammasome and downstream PGE2 signaling as well as an overproduction of IFNβ [57,134,135]. 

Differences in innate immunity between mouse strains seem to contribute to their susceptibility to TMEV-induced lesions. For instance, TMEV-DA-infected *SJL* mice show a significantly greater CNS expression of TLR2, 7, and 9 compared to *C57BL/6* mice and an upregulation of TLR3, 6, and 8, which was not detected in *C57BL/6* mice [136]. Interestingly, TMEV-IDD can be induced in normally resistant *C57BL/6* mice by stimulating TLR4 with LPS due to an upregulation of TLR7 and 9 [136]. *SJL* mice lack NK1.1^+^ and CD11c^int^ natural killer dendritic cells, which play a critical role in early CNS virus clearance [137]. Moreover, dendritic cells (DCs) from *SJL* mice are more permissive to viral infection and viral-induced apoptosis and produce higher levels of IFN-I and IFNγ than DCs from *C57BL/6* mice [138]. These cytokines can inhibit the maturation and function of DCs in a dose- and time-dependent manner. Consequently, a reduction in the number of mature DCs, which are necessary for T cell activation, in TMEV-BeAn-infected *SJL* mice seems to limit antiviral T cell responses and might result in pathogenic T cell responses [138]. Furthermore, *SJL* mice have a large number of innate immune CD11b^+^Ly6C^+^ myeloid-derived suppressor cells (MDSC) that can suppress virus-specific immune responses partly by nitric oxide production [109,139,140].

### 6.2. Expression of Pro-Inflammatory Mediators in Microglia, Macrophages, and Astrocytes

Mouse microglia express TLR1-9 and TMEV infection activates these cells to upregulate cytokines, chemokines, MHC class II, and costimulatory molecules, enabling microglia to efficiently present antigens to CD4^+^ T cells [141]. Several studies investigated the signaling pathways mediating the expression of pro-inflammatory mediators during a TMEV infection (Figure 6). Most TLRs signal via the activation of myeloid differentiation primary response gene 88 (MyD88), a Toll/IL1 receptor homology (TIR) domain-containing protein, which triggers TNF receptor associated factor (TRAF) 6 and TRAF3 to activate NF-κB and MAPK as well as IRF signaling, respectively [142]. In contrast, downstream signaling of TLR3 is mediated by the *TIR* domain-containing adaptor inducing IFNβ (TRIF) and not MyD88. Single-stranded TMEV RNA can be detected by TLR7, whereas dsRNA produced during the virus replication cycle is perceived by TLR3 and the Melanoma differentiation-associated gene 5 (MDA5) [143]. This RLR protein contains caspase activation and recruitment domains (CARDs), which interact with the downstream adaptor protein IFNβ promoter stimulator (IPS-1), also known as CARD adaptor inducing IFNβ (CARDIF), the mitochondrial antiviral-signaling protein (MAVS), and the virus-induced signaling adapter (VISA) [142]. As implicated by its name, MDA5 has a strong impact on the expression of IFN-I but not of IL6 and TGFβ by microglia and macrophages isolated from the brain of TMEV-BeAn-infected *129Sv* mice [143]. Mouse strain-specific variations in the expression of pro-inflammatory mediators also contribute to different reaction patterns in TMEV-IDD susceptible and resistant mice. *SJL* bone marrow-derived macrophages infected with TMEV exhibit increased virus RNA replication and infectious virus yields as well as greater IL6 production than *C57BL/6* and *B10.S* strain cultures [144]. The IL6 expression in TMEV-DA infected *C57BL/6* mice is partially controlled by IRF3 [145]. IRF3 is also involved in the maintenance of effective antiviral T cell memory responses and their granzyme B expression in response to a TMEV-DA infection [146]. IRF3 polymorphisms can explain changes in the expression of IFNβ, ISG56, IL6, and IL23 and the activation of apoptotic caspases. Nevertheless, these non-conservative mutations are absent from the IRF3 amino acid sequence of *SJL*, *C57BL/6*, and *B10.S* mice [144,147]. However, *SJL* and not *B10.S* macrophages exhibit constitutively active IRF-3, which causes increased IL12/23 p40 and decreased IL12 p35 levels in TMEV-DA-infected peritoneal macrophages obtained from this TMEV-IDD susceptible mouse strain [148]. Higher levels of IL6 and IL23 compared to IL12 in SJL mice favor a dominance of pro-inflammatory Th17-mediated responses over antiviral Th1-mediated responses contributing to virus persistence and predisposing to autoimmune diseases (see Section 7, Adaptive Immunity) [149,150,151,152].

**Figure 7:**
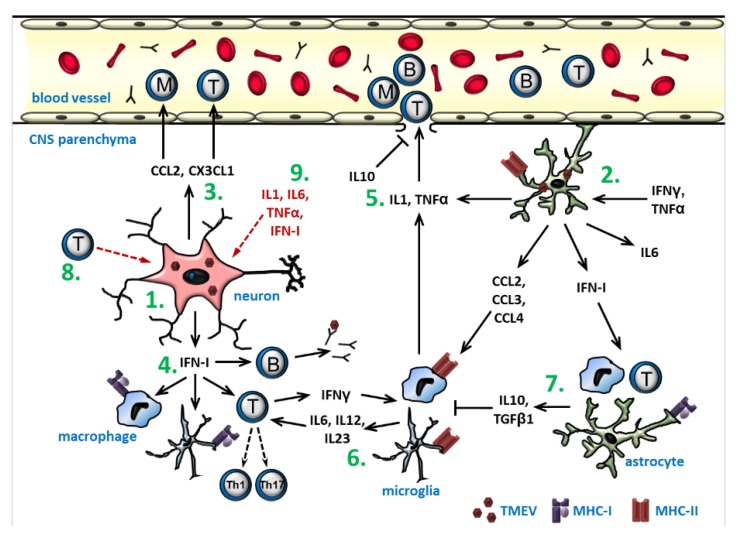
A schematic overview of the cytokine- and chemokine-mediated effects during early inflammation in a Theiler’s murine encephalomyelitis virus infection: (**1**) TMEV-infection of neurons results in the expression of chemokines and IFN-I. (**2**) Virus replication in astrocytes and their stimulation by IFNγ and TNFα also causes cytokine and chemokine production. (**3**) The chemokines CCL2 and CX3CL1 attract monocytes and lymphocytes. (**4**) IFN-I activates B cells, T cells, microglia, and macrophages and promotes antigen presentation. (**5**) IL1 and TNFα stimulate leukocyte extravasation, whereas IL10 stabilizes the blood–brain-barrier. (**6**) Macrophages and microglia activated by IFNγ and chemokines upregulate MHC class II and costimulatory molecules and secrete IL6, IL12, and IL23 to induce the differentiation of T cells into Th1 and Th17 cells, which have antiviral and pro-inflammatory properties. (**7**) The activation of macrophages and microglia is inhibited by the anti-inflammatory cytokines IL10 and TGFβ1, which the production of can also be induced by IFN-I. (**8**) The virus elimination is partly dependent on the activated cytotoxic T cells, which also cause neuronal necrosis and axonal injury. (**9**) Pro-inflammatory cytokines also have an ictogenic effect on neurons, predisposing to seizure development. The scheme summarizes the main results of multiple studies and does not include all the interactions demonstrated so far.

In contrast to microglia, resting astrocytes express high constitutive levels of TLR3 but no or very low levels of TLR7 and TLR8 [153]. Moreover, TLR3 stimulation by poly (I:C) results in stronger TLR1-6, IL6, TNFα, IFN-I, and iNOS expressions in astrocytes than stimulation by IFNγ and TNFα [153]. Nevertheless, IFNγ and TNFα but not poly (I:C) stimulation induces the upregulation of MHC class II and the functional ability of astrocytes to activate CD4^+^ T cells [153]. Therefore, astrocytes have to be infected by TMEV and stimulated by pro-inflammatory cytokines to be activated to the maximum level. Astrocytes are the main source of IFNβ in TMEV-GDVII-infected *C57BL/6* mice [154]. Despite stronger IL6 production in *SJL* than *C57BL/6* macrophages (see above), *C57BL/6* primary astrocytes produce higher levels of many cytokines (IL6 and TNFα), chemokines (CCL2 (MCP1), CCL3 (MIP1α), CCL4 (MIP1β), CCL5 (RANTES), CXCL1 (Gro1), and CXCL10 (IP10)), and adhesion molecules (ICAM-1 and VCAM-1) in response to TMEV-BeAn infections or stimulation with IFNγ and TNFα or poly (I:C) compared to *SJL*. In addition, TMEV-BeAn induces MHC class I molecules more effectively on *C57BL/6* than *SJL* astrocytes, showing an increased ability to present antigens to CD8^+^ T cells [155]. In contrast, TMEV-BeAn induces CXCL2 (MIP2) in astrocytes from *SJL* but not from *BALB/c* mice [156]. Similarly, demyelinating spinal cord lesions of *SJL* but not *BALB/c* mice contain CXCL1 [157]. Interestingly, the lack as well as the excessive presence of CXCL1 during TMEV-BeAn infections can increase the extent of demyelinating lesions [158]. The induction of cytokines (IL6 and TNFα), chemokines (CCL2 and CXCL10), and IFN-I genes in primary *C57BL/6* astrocytes following a TMEV-BeAn infection can be mediated by TLR3 but not TLR7 or other MyD88-mediated pathways [159]. The TLR3 signal also causes an upregulation of TLR2, which participates in the expression of IL1β, IL6, CCL2, and CCL5 genes by primary *C57BL/6* astrocytes following a TMEV-BeAn infection [160]. Nonetheless, PKR is important for the production of IFN-I, IL6, CCL2, and CXCL10 in primary SJL astrocyte cultures, whereas TLR3 plays only a minor role in the responses to a TMEV-BeAn infection [161]. Furthermore, astrocytes and oligodendrocytes isolated from MDA5^−/−^ *129Sv* mice show a reduced IFNβ expression [143]. Summing up, in vitro studies revealed that TLR-, MDA5-, and PKR-mediated pathways are induced by a TMEV infection, but their respective impacts on the expression of different effector proteins seems to be dependent on the specific cell type as well as the virus strain and genetic background of the mice. The main effects of the cytokines and chemokines produced during the early inflammatory processes in the CNS are shown in Figure 7.

### 6.3. Innate Immunity Accounts for Seizure Development during Theiler’s Murine Encephalomyelitis Virus Infection of C57BL/6 Mice

Several TMEV strains (GDVII, WW, BeAn) can induce seizures in *C57BL/6* mice to various degrees [51,96]. A recent study demonstrated that the severity of hippocampal neurodegeneration, the number of activated microglia/macrophages, and the ISG15 expression almost perfectly discriminate seizing from non-seizing *C57BL/6* mice after a TMEV-DA infection despite the fact that strong microglia/macrophage activation and some hippocampal damage are also present in *SJL* mice [23]. Limited hippocampal damage in TMEV-BeAn-infected *SJL* mice might be caused by the induction of IL10 receptor signaling, which exhibits anti-inflammatory and neuroprotective properties [162,163,164]. No difference between *SJL* and *C57BL/6* mice can be found in the TGFβ1 gene expression during acute polioencephalomyelitis, but seized *C57BL/6* mice show a strong TGFβ expression in hippocampal neurons, which is not present in unseized *C57BL/6* mice or mock- and TMEV-DA-infected *SJL* mice [2,164]. However, future studies are needed to elucidate the role of the anti-inflammatory cytokine expression in different mouse strains for the development of brain lesions and seizures. Interestingly, the H101 mutant of the DA strain, which is characterized by mutations in the VP1 loop II, the 5′ untranslated region, and the capsid protein-coding region, induces seizures and causes 100% mortality but only minor neuropathological changes [96,165]. Thus, elevated levels of pro-inflammatory cytokines, rather than neuronal cell death, play a dominant role in seizure induction. Similarly, an absence of overt neuronal loss was found in corneal kindled *C57BL/6* mice, which exhibit saturated dentate gyrus long-term potentiation and associated memory deficits [166].

The main mediators of acute inflammation are IL1β, IL6, and TNFα, which cause leukocyte extravasation, fever, production of acute phase proteins, and neuronal hyperexcitability [94,167]. IL1β and TNFα inhibit glutamate uptake into astrocytes, increasing the excitatory effect of this neurotransmitter [168]. Interestingly, changes in the astrocytic expression of the metabotropic glutamate receptor 5 (mGluR5) are consistently observed in epilepsy patients and animal models of epilepsy [169]. IL6 can shift the balance between synaptic inhibition and excitation in favor of the latter, most likely by reducing the number of inhibitory gamma-aminobutyric acid (GABA) receptors, changing the expression and function of glutamate receptors, and modulating sodium ions and calcium channels [94,170]. Moreover, transgenic mice with increased astrocytic IL6 production develop severe neurological diseases, which is associated with a progressive decline in avoidance learning, a profound increase in sensitivity to glutamatergic- but not cholinergic agonist-induced seizures, and concomitant degenerative changes in parvalbumin^+^ hippocampal interneurons, a specific subset of GABAergic inhibitory neurons [171,172,173]. In addition to these cytokines, the glial production of IFNβ also modulates the hippocampal network excitability, predisposing to the development of seizures [174].

In TMEV-DA-infected mice, IL1β does not seem to play a major role in seizures because IL1 receptor I- and MyD88-deficient *C57BL/6* mice have a seizure frequency similar to wild type mice. In contrast, only 10% of TNF receptor I- and 15% of IL6-deficient *C57BL/6* mice showed signs of seizure activity, and fewer seizures are seen in TNFα^−/−^ mice bred on a *C57BL/6* background [98,99,100,101,102,103,104,105,106,107,108,109,110,111,112,113,114,115,116,117,118,119,120,121,122,123,124,125,126,127,128,129,130,131,132,133,134,135,136,137,138,139,140,141,142,143,144,145,146,147,148,149,150,151,152,153,154,155,156,157,158,159,160,161,162,163,164,165,166,167,168,169,170,171,172,173,174,175]. Furthermore, *C57BL/6* mice with seizures have higher TNFα and IL6 mRNA brain levels compared to non-seizing mice [98]. The fact that TMEV-DA-infected IL6 knockout mice treated with recombinant IL6 also developed seizures demonstrated the ability of pathological IL6 levels in the periphery to cause seizures [176]. In addition to pro-inflammatory cytokines, the complement component C3 participates in the induction of acute seizures following the TMEV-DA infection of *C57BL/6* mice [177]. This component of the complement system is increased in the brain, especially in infiltrating macrophages and activated microglia [178]. Nevertheless, the exact mode of action of C3 in the development of recurrent and spontaneous seizures has not been elucidated so far. However, both resident and infiltrating myeloid cells seem to be involved in the seizure development, whereas T cells and NK cells only play a minor role [23,51,93,98,179]. In general, there are several lines of evidence that the adaptive immune system is not primarily involved in the occurrence of seizures following DA infection since (i) seizure occurrence correlates with the number of infiltrating macrophages but not lymphocytes; (ii) transgenic OT-I (CD8^+^, ovalbumin-specific TCR) mice, which do not develop TMEV-specific CD8^+^ T cell responses because the majority of CD8^+^ T cells is directed against ovalbumin, have a seizure occurrence similar to wildtype animals; and (iii) seizures and hippocampal damage occur very early on in TMEV infections, before the onset of overt adaptive immune responses [5,23,51,98,180].

In TMEV-DA-infected *C57BL/6* mice, TNFα and IL6 are predominantly produced by microglia and infiltrating macrophages, respectively [180]. Two different receptors mediate TNFα signaling and provoke antagonistic effects during the acute seizure period. TNF receptor (TNFR) 1 modulates glutamate receptor trafficking, leading to an increased excitatory synaptic transmission, whereas TNFR2 may inhibit limbic hyperexcitability [175]. Furthermore, a significant increase in the ratio of TNF receptors (TNFR1:TNFR2) in the hippocampus found after the TMEV-DA infection suggests that the microglial production of TNFα is involved in seizure initiation [175]. Nevertheless, the presence of microglia is also required to limit virus replication and spread, most likely by modulating T cell activation, and to prevent subsequent hippocampal damage and seizure development [181]. Studies using the treatment of *C57BL/6* mice with the tetracycline antibiotic minocycline or the flavanoid wogonin, both of which limit infiltration of immune cells into the CNS and their activation, indicate that infiltrating macrophages contribute to the development of acute seizures [180]. Similarly, the specific depletion of macrophages by the systemic administration of clodronate liposomes significantly reduced the number of seizing mice but did not reduce hippocampal damage or microglia activation in the hippocampus [182]. Thus, IL6 expression by infiltrating macrophages seems to contribute to seizure induction, whereas hippocampal damage might be predominantly mediated by TNFα released from activated microglia. Macrophage infiltration into the CNS of TMEV-DA-infected *C57BL/6* mice is dependent on the expression of CCL2 by neurons, which controls the recruitment of CCR2^+^ inflammatory monocytes during the most acute stage of encephalitis [183]. However, the knockout of CCR2 in *C57BL/6* mice results only in a moderate decrease in seizure severity, indicating that CCR2-independent CNS inflammatory mechanisms trigger the development of seizures during viral encephalitis [184]. This study also confirmed that both CCR2 and CX3CR1 play a role in the activation and proliferation of myeloid cells in the CNS, leading to neuronal loss, but are not necessary for viral clearance and seizure induction.

### 6.4. Innate Immunity Participates in Demyelination Processes and Cardiac Damage

Microglia and macrophages play a crucial role in the initiation and progression of TMEV-induced demyelination as effector cells and a source of immunomodulatory mediators [21,54,185,186]. The onset of virus-induced demyelination is associated with a dominating polarization of macrophages into pro-inflammatory M1 subtypes, while mounting M2 polarization together with continuous high M1-related gene expression can be found during the chronic-progressive phase [185]. Therefore, the perpetuating interaction between virus and macrophages/microglia results in the sustained expression of pro-inflammatory cytokines from M1-type cells, which fosters delayed type hypersensitivity, bystander demyelination, and disturbed myelin repair in TMEV-infected mice [185].

BeAn-infected *SJL* mice express several genes involved in the innate immune responses (Tlr7, Tlr8, Tlr9, Myd88, Isg15, Isg20, Mx1, and IL6) in the spinal cord during the late phase of TMEV-IDD at 165 days post infection [187]. In contrast, an increased expression of several mediators of adaptive immune responses, including IL13, TGFβ, and IFNγ, and the transcription factor forkhead box P3 (Foxp3) can be found already at the beginning of demyelination at 30 days post infection. The strong upregulation of the pro-inflammatory cytokines IL1α, IL6, and TNFα in the late phase of TMEV-IDD was also demonstrated in the cerebrospinal fluid [187]. Similarly, microarray analyses revealed a peak expression of the innate immunity genes, including genes involved in antigen processing and presentation, at 98 days post infection [87]. Consequently, adaptive immune responses seem to be involved in the initiation of demyelination, whereas innate immune pathways also contribute to the progression of the disease. Interestingly, despite an increased gene expression of ISG15 and PKR, the protein expression of these ISGs seems to be impaired in TMEV-BeAn-infected *SJL* mice and mainly restricted to demyelinated lesions during the late phase of TMEV-IDD. Moreover, *C57BL/6* mice exhibit higher ISG protein levels in the spinal cord than SJL mice during the disease. Therefore, high levels of antiviral IFN-I-dependent proteins in the spinal cord of *C57BL/6* mice might inhibit virus replication and contribute to the resistance of this mouse strain to TMEV-IDD [135]. In *C3H* mice, TMEV replication in the heart leads to an early upregulation of innate immune molecules, such as interferon-induced genes and chemokine genes by resident cells, which contributes to the recruitment of pro-inflammatory T cells and myocardial lesion development [5,105,188].

### 6.5. Modulation of the Cannabinoid System

The beneficial effects of Δ^9^-tetrahydrocannabinol (Δ^9^-THC) and cannabidiol (CBD) observed in MS therapy were recently studied in TMEV-DA-infected *SJL* mice [189]. These phytocannabinoids improve motor activity, reduce CNS inflammation and damage, and are associated with reduced vascular cell adhesion molecule (VCAM)-1 immunostaining and IL1β gene expression. CBD acts through peroxisome proliferator-activated receptor (PPAR) γ, whereas cannabinoid (CB) receptors are used for Δ^9^-THC signaling. VCE-004.8, a CBD quinone derivative, upregulates the expression of hypoxia-inducible factor (HIF)-dependent genes such as erythropoietin and vascular endothelial growth factor (VEGF)-A; induces angiogenesis; enhances the migration of oligodendrocytes; blunts IL17-induced M1 polarization; and inhibits LPS-induced COX-2 expression and PGE2 synthesis [190]. VCE-004.8 treatments of TMEV-DA-infected *SJL* mice also prevent demyelination, axonal damage, and immune cells infiltration [190]. Moreover, a selective CB2 agonist isoxazole derivative dampens neuroinflammation by reducing the microglial activation in the acute phase of TMEV-IDD [191]. Similarly, 2-arachidonoylglycerol, the major CNS endocannabinoid, limits acute neuroinflammation by modulating microglia and promoting MDSCs, reduces proteoglycans, and enhances remyelination in TMEV-IDD [192,193]. 

## 7. Adaptive Immunity in Theiler’s Murine Encephalomyelitis Virus Infection

### 7.1. CD4^+^ T Cells

Antigen-presenting cells within lymphoid organs and inflammatory lesions present self or pathogen antigens via MHC class ΙΙ molecules to naïve CD4^+^ T cells. Based on the functional properties and their cytokine profile, CD4^+^ T cells can be divided into Th1, Th2, Th17, and regulatory T cell subsets [194]. In TME, the beneficial and detrimental effects of the CD4^+^ T cell population have been described. CD4^+^ T cells are required for protective antiviral immunity, since CD4 knockout has been shown to cause virus persistence in TMEV-IDD resistant *C57BL/6* mice [195]. While pro-inflammatory Th1 responses account for myocardial damage in *C3H* mice following an infection [5,105,188], the adaptive immune responses seem to have only limited effects on the TMEV-induced seizure development in *C57BL/6* mice [5,23,51,98,180]. In chronic TME, virus-specific CD4^+^ T cells contribute to the disease progression via Th1-mediated delayed type hypersensitivity in the spinal cord [20,108,196]. The initial antiviral responses are supposed to result in bystander myelin damage and in the generation of myelin-specific CD4^+^ primed by epitopes spreading during the chronic phase [82,197]; however, the impact of the autoreactive CD4^+^ T cells on the disease progression and demyelination in infected mice remains unclear [198]. Recent studies have focussed on the interplay of CD4^+^ T cell subsets and its effects on the pathogenesis of TMEV infection. While the Th1/Th2 balance is seemingly not responsible for mouse strain-specific differences of TMEV-IDD susceptibility [199], skewed polarization of Th1/Th17 cells and a disproportional increase in regulatory T cells (T_reg_) have been shown to account for virus persistence in SJL mice [151,164,200,201,202,203].

#### 7.1.1. Regulatory T Cells

T_reg_ are a CD4^+^ T cell subset with immunomodulatory and suppressive properties, mediated by several mechanisms including soluble (IL10, IL35, and adenosine) and cell-associated (CTLA-4, CD39/CD73, and CD25) factors [204]. T_reg_, characterized by the expression of Foxp3, maintains immunological tolerance and prevents immunopathology in primary autoimmune disorders (e.g., EAE). However, in viral diseases, T_reg_ can exhibit dual functions: beneficial effects with reduced immune-mediated tissue damage and detrimental effects with disease exacerbation and viral persistence due to immunosuppression [202]. Following a TMEV-BeAn infection, the rapid activation and expansion of T_reg_ have been observed in the brain and spleen of *SJL* mice but not in *C57BL/6* mice, leading to the hypothesis that an imbalance between T_reg_ and effector T cells during acute infections contributes to virus persistence in TMEV-IDD susceptible mouse strains [164,201]. An expansion of natural T_reg_ specific for self-antigens might mistakenly recognize a structurally similar TMEV antigen (regulatory mimicry), which results in the excessive suppression of CD8-mediated antiviral responses. Since no differences regarding the inhibitory capacity were observed between TMEV-IDD resistant and susceptible mouse strains, quantitative rather than qualitative differences in T_reg_ responses might occur in TME [201].

T_reg_ depletion before a TMEV-BeAn infection using anti-CD25-antibodies results in increased CD4^+^ and CD8^+^ T cell responses, which reduces the viral load and delays the onset of clinical diseases [201]. The application of ex vivo induced T_reg_ (iT_reg_) to *SJL* mice prior to a TMEV-DA infection decreases the CNS recruitment of immune cells, resulting in enhanced virus replication and deterioration of acute clinical symptoms. By contrast, the transfer of iT_reg_ during the chronic disease phase ameliorated the demyelination by the IL10-mediated suppression of immunopathology [204]. The results show that T_reg_ represents a double-edged sword in TMEV-IDD susceptible mice with opposing effects depending on the disease phase. Interestingly, the treatment of TMEV-infected SJL mice with glatiramer acetate, an immunomodulatory drug used for MS patients, leads to T_reg_ expansion and increases IL10 production without the suppression of protective antiviral immunity [9].

In contrast to SJL mice, a limited role of T_reg_ has been shown in the pathogenesis of acute polioencephalomyelitis and antiviral immunity in TMEV-IDD resistant mice. The specific depletion of T_reg_ can be achieved using “depletion of regulatory T cell” (DEREG) transgenic mice, which bears the sequence for the diphtheria toxin receptor under the control of the foxp3 promotor [205]. Foxp3-depletion in DEREG mice prior to TMEV infection leads to an enhanced CNS recruitment of IFNγ-producing T cells but does not influence the virus load and acute hippocampal pathology [206]. Similarly, neither the adoptive transfer of ex vivo generated T_reg_ nor the expansion of endogenous T_reg_ or the antibody-mediated T_reg_-depletion sustainably impacts the virus replication in *C57BL/6* mice [201,203,206]. Interestingly, the T_reg_-expansion in CD8-deficient *C57BL/6* mice leads to chronic infection and spinal demyelination, demonstrating a modulatory function of T_reg_ on antiviral immunity, overridden by the dominating cytotoxic T cell responses in TMEV-IDD resistant mouse strains [207].

#### 7.1.2. Th17 Cells

Th17 cells are characterized by the lineage-specific transcription factor retinoic acid related orphan receptor (ROR) γt [5]. In TMEV infection, Th17 cells develop in an IL6-dependent manner in vitro and in vivo and secrete IL17, IL22, and TNFα [151]. IL6 overexpressing resistant mice show elevated IL17 responses, virus persistence, and demyelination, while IL6 knockout mice fail to develop Th17 cells in the CNS [150,152]. Interestingly, the gene for IL22 lies within the TMEV susceptibility locus Tmevp3 and has been proposed as a candidate gene for viral persistence [208]. The TMEV-BeAn infection induces strong Th17 responses in *SJL* mice, while C57BL/6 mice have negligible IL17 levels and robust Th1 responses [151]. *SJLxC57BL/6* F1 crosses show intermediate Th17 responses and CNS viral loads between those observed in *SJL* and *C57BL/6* mice [209]. Moreover, a blockade of IL17 boosts cytotoxic T cell functions and augments virus clearance in *C57BL/6* mice rendered susceptible to TMEV-IDD by LPS administration [151]. IL17 was shown to upregulate the antiapoptotic molecules (Bcl-2, Bcl-xL) in antigen-presenting cells, which protects them from Fas-mediated T cell cytotoxicity, thereby favoring virus persistence [150,151,210]. These data support the assumption that the balance between the pathogenic Th17 cell responses and the protective virus-specific CD8^+^ T cells determine the disease outcome in TME.

### 7.2. CD8^+^ T Cells

CD8^+^ T cells recognize the viral antigens via MHC class I, leading to cytotoxicity. In the TME model, protective and pathogenic effects of CD8^+^ T cells have been described, depending on the used mouse strain, CNS compartment, and disease phase. The resistance of *C57BL/6* mice to the development of demyelination is linked to the H-2D MHC class I locus [25,28,211,212,213], enabling vigorous MHC class I-restricted antiviral CD8^+^ T cell responses. This is exemplified by the transgenic introduction of the Db class I molecule in susceptible FVB mice (FVB/D^b^), resulting in robust antiviral cytotoxicity, TMEV elimination, and resistance to TMEV-IDD [214]. Similarly, the genetic ablation of CD8^+^ T cells in mice with a *C57BL/6* background has been shown to reduce antiviral immunity, which leads to TMEV persistence and demyelination in the spinal cord [195,215,216]. Moreover, genetic β_2_-microglobulin (β_2_M) deficiency, which results in disturbed MHC class I-restricted CD8^+^ T cell responses, also predisposes resistant mouse strains to develop prolonged TMEV infection and myelin loss [215,216].

CD8^+^ T cells of *C57BL/6* mice recognize TMEV capsid epitopes, including the immunodominant epitope VP2_121–130_ and two subdominant epitopes VP2_165–176_ and VP3_110–120_ [217,218]. TMEV-specific CD8^+^ T cells produce IFNγ and efficiently lyse virus-infected target cells [219]. Comparative analyses revealed that cytotoxic responses in the CNS of TMEV-IDD susceptible *SJL* mice are qualitatively similar to those of C57BL/6 mice with respect to the avidity to epitopes, IFNγ production, and cytolytic efficiency. However, probably due to an enhanced IL2 receptor signaling, higher quantities of virus-specific CD8^+^ T cells are present in the TMEV-IDD resistant strains during the initial infection phase [220]. Following acute polioencephalomyelitis in *C57BL/6* mice and the TMEV elimination from the brain, effector CD8^+^ T cell populations are contracted to avoid continuous immunopathology. Effector T cells are replaced by tissue resident memory CD103^+^ CD69^+^ CD8^+^ T cells, which reside in the CNS, as demonstrated in a TMEV-DA infection. This switch is mediated by the immune checkpoint ligand B7-H1 (PD-L1) and has been shown to protect from virus re-exposure in TMEV-IDD resistant mice [221].

Besides protecting from viral infection, cytotoxicity has the ability to cause detrimental effects and contributes to acute neuropathology in TME. Virus-specific CD8^+^ T cells target the brain grey matter, including the hippocampus, in acutely infected *C57BL/6* mice and form immune synapses with infected neurons. The engagement of neurons by CD8^+^ T cells and subsequent granzyme B release supposedly mediates axonal and neuronal damage in these animals [222]. Referring to this, using magnetic resonance imaging, virus-specific cytotoxic CD8^+^ T cells have been shown to induce T1 hypointensive lesions (“T1 black holes”) in mouse brains following a TMEV-DA infection, as demonstrated by adoptive transfer experiments in RAG-1-deficient mice and epitope-specific CD8^+^ T cell depletion experiments in *C57BL/6* mice [223]. Moreover, MHC class I-restricted cytotoxicity towards viral epitopes contributes to brain atrophy and hippocampal neuronal loss in *FVB/D^b^* mice, demonstrating a direct pathogenic role of CD8^+^ T cells during an acute TMEV infection [214]. Strengthened antiviral CD8-mediated cytotoxicity boosted by intravenous administration of the immunodominant VP2_121–130_ peptide also leads to excessive, fatal neuroinflammation with perforin-mediated blood–brain-barrier disruptions and CNS microhemorrhages in *C57BL/6* mice infected with TMEV-DA [224,225,226]. However, the impact of cytotoxicity on seizure development in TMEV-infected mice remains elusive [5,23,51,98,180]. 

Similar to TMEV-IDD resistant mice, both the protective and pathogenic effects of CNS-infiltrating CD8^+^ T cells can be observed in mouse strains prone to developing persistent TMEV infections and demyelination. The thymectomy of TMEV-IDD susceptible *SJL* and *CBA* mice leads to reduced CD8^+^ T cell numbers with an early disease onset and profound clinical signs upon a TMEV-BeAn infection [227]. Moreover, the antibody-mediated depletion of CD8^+^ T cells prior to a TMEV infection has been shown to diminish viral clearance causing an increased severity of demyelinating diseases in susceptible *SJL* mice (TMEV-BeAn) and the presence of small demyelinating lesions in resistant *C57BL/10* mice (TMEV-DA), respectively [227,228]. The adoptive transfer of CD8^+^ T cells specific for virus capsid proteins during the acute infection phase results in virus elimination and prevention from demyelinating diseases in susceptible mice infected with TMEV-BeAn [212,229]. The protective effects of CD8^+^ T cells were also proven by experiments using β_2_M-deficient *SJL* mice, showing higher virus concentrations compared to wild type controls and an association with increased macrophage infiltration and pro-inflammatory cytokine production in the spinal cord upon a TMEV-BeAn infection [230]. By contrast, the preserved motor neuron function in CD8^+^ T cell-depleted mice and reduced neurological defects in MHC class I-deficient mice have been described [215,231]. Similarly, the ablation of antiviral CD8^+^ T cells in IFNγ receptor-depleted mice results in the preservation of motor neuron functions and in the maintenance of axonal transport mechanisms following a TMEV-DA infection, indicating that cytotoxic T cells might be responsible for the initiation of axon injury following demyelination [232]. In TMEV-induced demyelinating lesions, MHC class I are expressed on axons, while MHC II are predominately present on cell bodies in the spinal cord [233]. The perforin release by activated CD8^+^ T cells seems to be involved in axon damage, since perforin-deficient mice show the preservation of demyelinated axons and motor function despite virus persistence and demyelination [233,234,235]. During the late chronic demyelination phase, the deletion of CD8^+^ T cells directed against virus capsid proteins VP1 and VP2 have been shown to enhance remyelination and to prevent further myelin and axonal loss [236]. Similarly, the genetic deletion of β_2_M in B10.Q mice promotes Schwann cell- and oligodendrocyte-mediated remyelination on TMEV-DA infections, supporting the hypothesis that virus-specific CD8^+^ T cells limit naturally occurring neuroregeneration in TMEV-IDD susceptible mice [237].

Autoreactive CD8^+^ T cells, which have the ability to produce IFNγ and to lyse non-infected syngeneic target cells in a Fas-dependent manner, can be isolated from *SJL* mice infected with TMEV-DA. The adoptive transfer of these cells causes brain and spinal cord inflammation together with oligodendrocyte apoptosis in naïve mice [238,239]. The identification of dual or chimeric T cell receptors directed against viral and self-antigens might explain the development of autoimmunity by CD8^+^ T cells in TME [240]. As observed in the TMEV-BeAn infection of *SJL* mice, CD8^+^ T cells directed against the predominant VP3_159__–166_ epitope but not those directed against the subdominant epitopes display pathogenic roles, demonstrating different properties of epitope-specific CD8^+^ T cell subsets in the pathogenesis of TMEV-IDD [241,242]. Variations in the epitope recognition and avidity of CD8^+^ T cells have been found in SJL mice infected with the BeAn and DA strains of TMEV, which might account for virus strain-specific effects on neuropathology in TMEV-IDD susceptible mouse strains [243].

### 7.3. B Cells and Humoral Immunity

B cells play an essential role in innate and adaptive immunity in infectious disorders. Neutralizing antibodies directed against TMEV capsid proteins can be found in blood and cerebrospinal fluid of infected mice [20]. B cell depletion using anti-CD20-antibodies prior to or during acute TMEV-BeAn infection reduces the systemic and CNS humoral responses, which leads to an enhanced viral replication and the worsening of clinical disability. Data indicate that early antiviral antibody responses have the ability to delay disease progression in *SJL* mice [244]. In TMEV-IDD resistant *C57BL/6* mice, early protective antibody responses seem to be superseded by antiviral cytotoxicity since the protective effects of TMEV-specific antibodies can be found only in the absence of CD8^+^ T cells [245].

Gene expression analyses revealed an early activation of the genes involved in antigen presentation and B cell activation in cervical lymph nodes during an acute TMEV-BeAn infection in *SJL* mice [246]. Subsequently, antibody-secreting cells presumably migrate to the CNS. With the disease progression, the increasing neutralizing antibody titers with the accumulation of CD138^+^ plasma cells as well as the gene transcription involved in antigen presentation, plasma cell differentiation, and antibody production can be found locally in the spinal cord of *SJL* mice, suggesting intrathecal antibody production during the chronic demyelinating phase [87,247,248]. Interestingly, besides TMEV-specific antibodies, immunoglobulins, which have the ability to cross-react with myelin components, can be found in chronically infected mice [198,249]. Autoantibody formation and autonomous immune responses in the CNS behind an intact blood–brain-barrier are discussed to contribute to the disease progression in MS [249]. However, so far, the impact of the myelin-specific antibodies and the CNS-restricted humoral responses in the pathogenesis of TMEV-IDD remains elusive. Only a few B cells can be found in the hearts of *C3H* mice following an infection, indicating a minor role of humoral responses in the pathogenesis of TMEV-induced myocardial damage [188].

### 7.4. Lymphocyte Apoptosis of Immune Cells

Apoptosis of leukocytes plays a critical role in immune cell homeostasis and the termination of inflammatory responses in infectious and autoimmune CNS disorders. Brain-infiltrating CD3^+^ T cells in mice infected with the TMEV-DA undergo activation-induced cell death (AICD), which results in a resolution of inflammatory responses. The expression of Fas and Fas ligand (FasL) on CD3^+^ T cells suggests T cell receptor engagement leads to the AICD of infiltrating T cells during early acute TME. In contrast, during late chronic demyelinating disease and despite dense perivascular and leptomeningeal infiltrates, only very few immune cells undergo apoptosis in the spinal cord. A lack of Fas-mediated AICD of T cells is supposed to be a consequence of the increased expression of the apoptosis inhibitory molecule Bcl-2 [250]. Noteworthily, *SJL* mice infected with the BeAn-strain of TMEV exhibit a considerable amount of apoptotic CD3^+^ T cells also during the demyelinating phase, indicating ongoing AICD in the spinal cord [251]. However, time–course analyses of cell-specific apoptotic events clearly indicate a significant reduction in T cell apoptosis in the late chronic spinal cord lesions (196 days post infection) of *SJL* mice following an infection with TMEV-BeAn [57]. The decreased apoptotic cell death in the progressive phase of TME is associated with the expression of several factors involved in apoptosis inhibition and cell survival as determined by a microarray analysis. For instance, the increased transcription of pro-survival genes in the spinal cord of infected TMEV-infected *SJL* mice, such as adenosine deaminase, CD28, IL7 receptor (CD127), PARP-9, PARP-14, phosphoinositide-3-kinase catalytic subunit gamma (Pik3cg), and Bcl2-related protein A1d, is capable of inducing leukocyte apoptosis resistance and prolonged inflammation, as described for MS and/or EAE [57]. These data indicate that a failure of pathogenic T cell elimination by apoptosis might contribute to disease progression in TMEV-infected *SJL* mice.

## 8. Conclusion and Outlook

Disease susceptibility in TME depends on different factors such the age, sex, and genetic backgrounds of mice. Furthermore, the virus strain-specific properties and the infection course significantly contribute to the complexity of the TMEV-induced lesion development. The pathology of the virus infections in humans and animals is caused by direct lytic effects and excessive inflammation leading to immune-mediated damage. Particularly in immune privileged organs, such as the CNS, pathogens have the ability to trigger autoimmune diseases by the bystander activation of autoreactive cells, molecular mimicry, and the breakdown of immune tolerance. Epidemiological studies provide evidence that viruses participate in the pathogenesis of MS in predisposed individuals. Similarly, uncontrolled antiviral immunity against cardiotropic viruses leads to chronic autoimmune myocarditis. Inflammatory responses also account for acute seizures in viral encephalitis, increasing the risk of developing epilepsy with chronic spontaneous seizures. The prevention of immune-mediated tissue damage is particularly important in organs which have only limited regeneration capacities, such as the CNS and heart. Therefore, therapeutic approaches to selectively reduce autoimmunity and immunopathology while maintaining protective antiviral immunity are needed in diseases with an infectious etiology or that develop in parallel with an infectious agent. The variety of transgenic mouse models and genetically engineered viruses in TME studies provide novel insights into organ-specific immunopathology and the balance of inflammatory responses in viral diseases. In addition, the TME model enables the investigation of regenerative strategies, including stem cell-based approaches for the treatment of demyelinating diseases [252,253,254]. With respect to viral-induced seizures and epilepsy, the discovery and characterization of new therapeutics to prevent, ameliorate, and inhibit seizures/epilepsy are in urgent need because seizures are often refractory to current treatments and associated with comorbidities such as psychiatric disorders and cognitive decline [90]. Besides the inflammatory pathways as targets for intervention, the recent finding that positive modulation of the mGluR5-reduced seizures and the percent of microglia and macrophages producing TNF-α in TMEV-infected *C57BL/6* mice [255] indicates that mGluR5 may represent a potential therapeutic target. Holistic approaches, such as global gene expression analyses comparing different mouse strains and the use of congenic mouse models, will help to identify targets responsible for persistent infection and disease susceptibility.

## Abbreviations

AICD	Activation-induced cell death
AP-1	Activator protein 1
β_2_M	β_2_-Microglobulin
CARD	Caspase activation and recruitment domain
CARDIF	CARD adaptor inducing IFNβ
CB	Cannabinoid
CBD	Cannabidiol
CLR	C-type lectin receptor
CNS	Central nervous system
DAMP	Damage-associated molecular pattern
DC	Dendritic cell
Δ^9^-THC	Δ^9^-Tetrahydrocannabinol
DEREG	Depletion of regulatory T cells
dsRNA	Double-stranded RNA
EEG	Electroencephalogram
eIF2α	Eukaryotic translation initiation factor 2α
FADD	Fas-associated protein with death domain
FasL	Fas ligand
GABA	Gamma-aminobutyric acid
HIF	Hypoxia-inducible factor
ICE	IL1β converting enzyme
IFN-I	Type I interferon
IL	Interleukin
IPS-1	IFNβ promoter stimulator
IL	Interleukin
IRES	Internal ribosomal entry site
IRF	Interferon regulatory factor
ISG	Interferon stimulated gene
iT_reg_	Induced T_reg_
lncRNA	Long noncoding RNA
LPS	Lipopolysaccharides
MAPK	Mitogen-activated protein kinase
MAVS	Mitochondrial antiviral-signaling protein
MBP	Myelin basic protein
MDA5	Melanoma differentiation-associated gene 5
MDSC	Myeloid-derived suppressor cell
mGluR5	Metabotropic glutamate receptor 5
MHC	Major histocompatibility complex
MOG	Myelin oligodendrocyte glycoprotein
MS	Multiple sclerosis
MyD88	Myeloid differentiation primary response gene 88
NF-κB	Nuclear factor κ-light-chain-enhancer of activated B cells
NLR	NOD-like receptor
Nup	Nuclear pore protein
OAS	Oligoadenylate synthetase
ORF	Open reading frame
PAMP	Pathogen-associated molecular pattern
Pik3cg	Phosphoinositide-3-kinase catalytic subunit gamma
PKR	dsRNA-Activated protein kinase
PLP	Proteolipid protein
PPAR	Peroxisome proliferator-activated receptor
PRR	Pattern recognition receptor
RIG	Retinoic acid-inducible gene
RLR	RIG-I-like receptors
ROR	Retinoic acid related orphan receptor
Th	T helper
TIR	Toll/IL1 receptor homology
TLR	Toll-like receptor
TME	Theiler’s murine encephalomyelitis
TMEV	TME virus
TMEV-IDD	TMEV-induced demyelinating disease
TNF	Tumor necrosis factor
TNFR	TNF receptor
TRADD	TNFR1-associated death domain protein
TRAF	TNFR-associated factor
T_reg_	Regulatory T cells
TRIF	TIR domain-containing adaptor inducing IFNβ
VCAM	Vascular cell adhesion molecule
VEGF	Vascular endothelial growth factor
VHEV	Vilyuisk human encephalomyelitis virus
VISA	Virus-induced signaling adapter
